# A Priori Prediction of Breast Cancer Response to Neoadjuvant Chemotherapy Using CT Radiomics

**DOI:** 10.3390/cancers17162706

**Published:** 2025-08-20

**Authors:** Deok Hyun Jang, Laurentius O. Osapoetra, Lakshmanan Sannachi, Belinda Curpen, Ana Pejović-Milić, Gregory J. Czarnota

**Affiliations:** 1 Physical Sciences, Sunnybrook Research Institute, Toronto, ON M4N 3M5, Canada; 2 Department of Radiation Oncology, Sunnybrook Health Sciences Centre, Toronto, ON M4N 3M5, Canada; 3 Department of Physics, Toronto Metropolitan University, Toronto, ON M5B 2K3, Canada; 4 Department of Medical Biophysics, Faculty of Medicine, University of Toronto, Toronto, ON M5T 1P5, Canada; 5 Department of Medical Imaging, Sunnybrook Health Sciences Centre, Toronto, ON M4N 3M5, Canada; 6 Department of Radiation Oncology, Faculty of Medicine, University of Toronto, Toronto, ON M5T 1P5, Canada

**Keywords:** radiomics, CT, breast cancer, neoadjuvant chemotherapy, response prediction, machine learning

## Abstract

(1) Background: Response to neoadjuvant chemotherapy (NAC) is a key prognostic indicator in breast cancer. However, current response evaluation methods rely on histopathological assessment after surgery, delaying opportunities for early treatment adaptation. This study aimed to develop a machine learning model by integrating radiomic features extracted from pre-treatment, contrast-enhanced computed tomography (CT) images with baseline clinical variables to predict NAC response before therapy initiation. (2) Methods: The study investigated two categories of response: (i) pathologic complete response (pCR) versus non-pCR, and (ii) clinical response versus non-response, where clinical response was defined as a reduction in tumor size of at least 30%, encompassing both complete and partial responses. Radiomic features (*n* = 214) were extracted from intratumoral and peritumoral regions of pre-treatment CT images. Clinical variables (*n* = 7) were also incorporated to enhance predictive capability. A predictive model was developed using XGBoost algorithm, and performance was evaluated across ten independent data partitions using metrics including accuracy, precision, sensitivity, specificity, F1-score, and AUC. (3) Results: A total of 177 patients were enrolled in the study. The combined clinical-radiomic model set exhibited superior predictive performance compared to models based solely on either radiomic or clinical features. For pCR classification, integrating clinical and radiomic features produced the strongest model, achieving 82.8% accuracy with an AUC of 0.846. The clinical model alone reached 71.4% accuracy and an AUC of 0.797, while the radiomic model achieved 67.5% accuracy and an AUC of 0.615. For clinical response classification, the combined model again outperformed the individual models, achieving 71.7% accuracy with an AUC of 0.725, compared with 65.0% accuracy and an AUC of 0.666 for the clinical model, and 65.6% accuracy with an AUC of 0.615 for the radiomic model. (4) Conclusions: These results demonstrate that integrating CT radiomic features with clinical information enhances the prediction of NAC response, supporting the potential for earlier and more personalized therapeutic decision-making in breast cancer management.

## Simple Summary

Early prediction of response to neoadjuvant chemotherapy (NAC) can help clinicians tailor personalized treatment strategies for breast cancer patients. This study explored a machine learning approach that combined radiomic features, which are quantitative imaging characteristics extracted from routine pre-treatment CT scans, with standard clinical information including tumor size, patient age, nodal status, histologic grade, and receptor status to predict chemotherapy outcomes. The results indicated that integrating CT-based radiomic features with clinical variables significantly improved the overall predictive performance compared to clinical data alone. This combined approach may allow for earlier identification of patients who are less likely to benefit from standard chemotherapy, thereby supporting more personalized and effective breast cancer management.

## 1. Introduction

With the widespread adoption of mammographic screening and advances in therapeutic approaches, breast cancer mortality rates have steadily declined [1]. However, breast cancer remains a major global public health challenge, as it is the most commonly diagnosed cancer and the leading cause of cancer-related death among women worldwide [2]. Current clinical management of nonmetastatic breast cancer employs a multidisciplinary approach, integrating systemic therapies such as chemotherapy, endocrine therapy, and targeted agents, alongside local interventions including surgery and radiation therapy [3].

Neoadjuvant chemotherapy (NAC), a systemic treatment delivered before surgery, has become a cornerstone in the management of patients with locally advanced breast cancer (LABC) and inflammatory breast cancer. Moreover, NAC is increasingly employed in selected high-risk early-stage tumors, such as those with human epidermal growth factor receptor 2 (HER2)-enriched or triple-negative breast cancers [4]. The purpose of NAC is to reduce tumor volume, enhance the feasibility of breast-conserving surgeries, and improve overall surgical outcomes [5]. Beyond its role in tumor downstaging, NAC reveals tumor responsiveness to systemic treatment, serving as an important prognostic indicator [6]. Achievement of pathologic complete response (pCR), defined as the complete absence of invasive carcinoma in breast tissue and axillary lymph nodes following NAC [7,8], is strongly associated with improved long-term survival outcomes, especially in more aggressive breast cancer subtypes [9].

However, achieving pCR remains challenging, with considerable variability across breast cancer subtypes [8,9]. HER2-enriched and triple-negative breast cancers generally demonstrate higher pCR rates than luminal A and luminal B subtypes [8,9]. Clinically, partial response is the most common treatment outcome, characterized by residual tumor that exhibits an intermediate level of regression [10,11]. While pCR is associated with the most favorable long-term outcomes, partial response is linked to improved prognosis relative to stable or progressive disease [10,11]. Given the prognostic significance of NAC response, early identification of patients unlikely to achieve favorable outcomes could facilitate timely therapeutic adaptations that may improve survival outcomes. Currently, definitive assessment of response relies upon postoperative histopathological analysis, which limits its clinical utility for guiding early therapeutic decisions.

Radiomics has emerged as a promising tool to non-invasively characterize tumor biology by extracting high-dimensional quantitative features from standard radiologic images [12,13,14]. In the context of breast cancer, radiomics has been applied to a range of objectives, including tumor detection, molecular subtype identification, evaluation of treatment response, and prognostic assessment [15,16,17,18]. In addition, the use of multiple imaging modalities such as quantitative ultrasound (QUS) [19,20], computed tomography (CT) [21,22], and magnetic resonance imaging (MRI) [23,24,25,26] has been explored, each contributing unique and complementary information about tumor characteristics. While contrast-enhanced CT remains an integral component of standard breast cancer management, primarily used for staging after diagnosis, the application of CT radiomics to breast cancer is still relatively underexplored compared to other modalities. Given its widespread availability and ability to capture tumor morphology, CT-based radiomics holds considerable promise for predictive modeling. Moreover, because the physics behind CT image acquisition differs substantially from MRI and QUS, it has the potential to offer a unique radiomic perspective related to tumor characteristics and complement existing MRI- and QUS-based radiomic approaches.

This study aims to investigate the feasibility of predicting breast cancer response to NAC using machine learning by integrating clinical variables and pre-treatment CT radiomic features. Radiomic features from intratumoral and peritumoral regions were analyzed to comprehensively characterize the tumor and the surrounding microenvironment. Unlike previous CT-based radiomics studies that focused on a single response definition, this study evaluates two complementary response criteria: pCR versus non-pCR, and clinical response versus non-response, with clinical response encompassing both complete and partial response. Prior studies have demonstrated that classifying patients by clinical response provides prognostic insight, including differences in recurrence-free survival [19]. Thus, by incorporating both response criteria, this study aims to provide a more comprehensive evaluation of therapeutic effectiveness and enable earlier identification of patients at higher risk of unfavorable outcomes.

## 2. Materials and Methods

### 2.1. Patient Selection

This retrospective analysis reviewed cases from 2013 to 2019 at Sunnybrook Health Sciences Centre, Toronto, Canada, involving individuals with LABC or high-risk early-stage breast cancer who completed standard NAC. Patients received three cycles of fluorouracil, epirubicin, and cyclophosphamide, with subsequent administration of three cycles of docetaxel every three weeks. In an alternative dose-dense regimen, four cycles of doxorubicin and cyclophosphamide were given, with subsequent administration of four cycles of paclitaxel every two weeks. HER2-positive patients additionally received trastuzumab concurrently as targeted therapy. Patients were also required to have undergone a biopsy and contrast-enhanced MRI and CT scans acquired as part of the diagnostic work-up prior to NAC, and surgery following NAC. The MRI scan was specifically required for pre-treatment tumor size assessment. Cases were excluded for inadequate quality CT images, significant artifacts, tumor extension beyond the CT field of view (FOV), incomplete biopsy or surgical pathology reports, or the presence of breast implants. Approval from the Institutional Research Ethics Board was obtained before conducting the study, and patient data were anonymized prior to analysis to maintain confidentiality.

### 2.2. Response Evaluation

In this study, tumor response was evaluated using two complementary criteria. Criterion 1 distinguished between pCR and non-pCR groups based on surgical pathology, with the latter group including both partial responders and non-responders. For this definition of pCR, the presence of ductal carcinoma in situ was not considered. Criterion 2 distinguished between responders and non-responders. To determine response, a modified grading system [20], adapted from the Response Evaluation Criteria in Solid Tumors (RECIST) guidelines [27], was applied, in which pre-treatment tumor size from MRI was compared with post-treatment tumor size determined from surgical pathology. This comparison between radiologically and pathologically assessed tumor size was employed because preoperative imaging after NAC was not routinely performed at the institution. Furthermore, postsurgical histopathology is considered the gold standard for evaluating treatment response. Under this criterion, a size reduction greater than 30% was defined as a response, encompassing both pCR and partial response. In contrast, a size reduction of less than 30% was considered non-response, including both stable and progressive disease. Tumors with residual cellularity below 1% were also considered responsive under this definition [20].

### 2.3. CT Acquisition and Analysis

Pre-treatment contrast-enhanced CT scans were obtained as part of the institutional standard of care for staging, using either a Brightspeed or Lightspeed CT scanner (GE Healthcare, Chicago, IL, USA). The images were acquired using a helical acquisition mode during the chest/abdomen/pelvis protocol. For both scanners, the imaging parameters included a tube voltage of 120 kVp, slice thickness of either 2.0 mm or 2.5 mm, a fixed matrix size of 512 × 512 pixels, and in-plane pixel size ranging from 0.5 to 1.0 mm, with Omnipaque 300 as the contrast agent.

**Figure 1:**
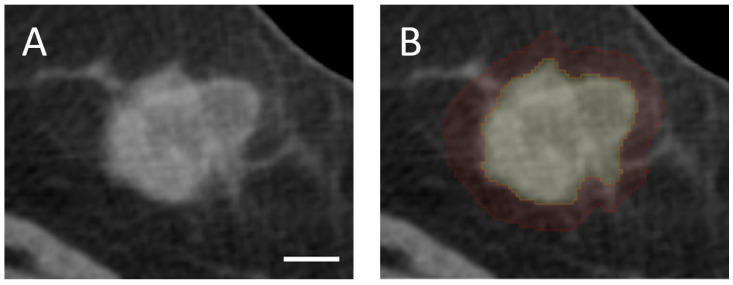
Example of tumor segmentations on pre-treatment contrast-enhanced CT images. (**A**) Contrast-enhanced CT image; (**B**) the same CT image with intratumoral (yellow) and peritumoral (red) segmentations. A scale bar corresponding to 1 cm is shown in the bottom right corner of image (**A**).

Tumor volume was delineated manually slice-by-slice in the axial plane using 3D Slicer (version 5.2.1), an open-source medical imaging software. Furthermore, a peritumoral segmentation was created by isotropically expanding the tumor boundary by 5 mm in all dimensions to capture peritumoral characteristics that have demonstrated clinical relevance [26,28]. The chest wall and skin were excluded from the peritumoral ROI to ensure that radiomic analysis is restricted to breast tissue. All segmentations were subsequently reviewed and validated by an expert radiologist and an expert oncologist. Representative examples of intratumoral and peritumoral segmentations are presented in Figure 1.

### 2.4. Radiomic Feature Extraction

The radiomic feature extraction pipeline was constructed using PyRadiomics (version 3.0.1), an open-source Python (version 3.8.8) library for radiomic analysis [29]. Two pre-processing steps were implemented to standardize image quality before feature extraction. First, CT images and their corresponding segmentations were resampled to an isotropic voxel size of 1 mm × 1 mm × 1 mm. Second, image intensities were discretized using a fixed bin width of 2. Intensity standardization was not performed to preserve the physical significance of Hounsfield Units (HU). Feature extraction was performed independently for each of the intratumoral and peritumoral segmentations.

Three categories of features were generated: 14 shape features, 18 first-order statistical features, and 75 second-order features. Shape features characterized the three-dimensional geometry and size of the segmentations, independent of voxel intensity [14]. First-order features described the statistical distribution of voxel intensities within each segmentation [14]. Second-order features, often referred to as texture features, quantified the spatial relationships between voxels of different intensities [14]. The 75 texture features included 24 derived from the Gray Level Co-occurrence Matrix (GLCM), 16 from the Gray Level Run Length Matrix (GLRLM), 16 from the Gray Level Size Zone Matrix (GLSZM), 14 from the Gray Level Dependence Matrix (GLDM), and 5 from the Neighboring Gray Tone Difference Matrix (NGTDM). A complete list of the extracted features is provided in Supplementary Table S1. Across both intratumoral and peritumoral regions, a total of 214 features were extracted per patient: 28 shape features, 36 first-order features, and 150 second-order features.

### 2.5. Clinical Features

Clinical data retrieved from the institutional electronic medical records were incorporated into the analysis to reflect key factors that guide treatment decisions in breast cancer management. Estrogen receptor (ER), progesterone receptor (PR), and HER2 status were included as binary features (categorized as positive or negative). Additional clinical variables comprised patient age, histological grade (categorized as G1, G2, or G3), tumor size (in millimeters), and clinical nodal status (categorized as N0 through N3). In total, seven clinical features were used for modeling.

### 2.6. Machine Learning Classification

To investigate whether integrating radiomic features could enhance the predictive capability provided by clinical data, three classification models were developed from three distinct feature sets: (i) a clinical set comprising seven variables, (ii) a radiomic set comprising 214 features, and (iii) a combined set encompassing all 221 clinical and radiomic features. As the identification of less-responsive patients holds greater clinical significance, non-responsive groups were labeled as positive, while responsive groups were labeled as negative.

Prediction models were trained and evaluated across 10 independent partitions. For each partition, patients were randomly allocated to training (80%) and hold-out test sets (20%), maintaining the original class proportions through stratified sampling. Feature selection, hyperparameter tuning, and model training were performed exclusively on the training set, and model performance was assessed on the corresponding test set. Performance was evaluated based on multiple metrics, including accuracy, precision, sensitivity, specificity, F1-score, and area under the receiver operating characteristic curve (AUC), with results averaged across the 10 independent partitions to provide a robust estimate of the overall model performance.

Prior to feature selection, ComBat harmonization was applied to adjust for scanner-specific differences, thereby reducing scanner-related variability in radiomic features [30]. Subsequently, the harmonized feature values underwent robust scaling for standardization and to minimize the influence of outliers. Notably, both pre-processing steps were applied after data partitioning to prevent information leakage. Feature selection was then implemented in two stages to reduce computational complexity and mitigate the risk of overfitting. Initially, the Minimum Redundancy Maximum Relevance (mRMR) algorithm identified features based on mutual information, selecting those most relevant to the target label and least redundant with each other [31]. The maximum number of features selected by mRMR was set to approximately 10% of the total sample size to minimize the risk of overfitting [32]. Subsequently, recursive feature elimination (RFE) was employed, using the Extreme Gradient Boosting (XGBoost) classifier (version 3.0.4) with 5-fold cross-validation within the training set. Feature selection was conducted independently within each partition, and the frequency of selection across the 10 iterations was recorded for each selected feature.

Final model training was conducted using the XGBoost classifier [33], a gradient-boosted tree ensemble method commonly employed in radiomic analyses due to its high predictive performance and computational efficiency [34,35]. Hyperparameter tuning was performed via grid search with 5-fold cross-validation within the training set. The hyperparameter settings are summarized in Supplementary Table S2. To address the class imbalance present in both response criteria, class weighting was applied in the XGBoost classifier by setting the “scale_pos_weight” parameter as the ratio of negative to positive samples within each training fold. This adjustment amplifies the importance of the minority class by penalizing its misclassification during training. Additionally, XGBoost can effectively handle class imbalance, as it trains in iterations and places greater emphasis on correcting misclassified samples from earlier stages, which contributes to improved recognition of underrepresented classes.

### 2.7. Statistical Analysis

Statistical analysis was conducted to compare the distributions of clinical parameters and selected radiomic features between responsive and non-responsive groups defined by the two criteria. Continuous variables were first assessed for normal distribution using the Shapiro–Wilk test. Depending on the results of this normality assessment, continuous variables were then analyzed using either the independent samples *t*-test or the Mann–Whitney U-test. Categorical variables were analyzed using Fisher’s exact test. Classification performances of the three feature sets were compared using paired two-tailed *t*-tests. Statistical significance was defined as *p* < 0.05.

## 3. Results

### 3.1. Patient Characteristics

A total of 177 patients met the inclusion and exclusion criteria and were included in the analysis. Clinical and pathological characteristics of the study cohort are presented in Table 1 for criterion 1 (pCR versus non-pCR) and Table 2 for criterion 2 (response versus non-response). Under criterion 1, 37 patients (20.9%) achieved pCR, whereas 140 patients (79.1%) had residual disease and were categorized as non-pCR. Under criterion 2, 124 patients (70.0%) were classified as responders, and 53 patients (30.0%) as non-responders.

Comparative analysis identified statistically significant differences between responsive and non-responsive groups under both criteria with respect to histologic grade and receptor status (ER, PR, and HER2). For criterion 1, the difference in nodal status was statistically significant as well, and the difference in initial tumor size approached statistical significance with *p*-value of 0.073. For criterion 2, the difference in age approached statistical significance with *p*-value of 0.064.

In terms of image acquisition, 127 patients were scanned using a GE Lightspeed scanner and 50 patients using a GE Brightspeed scanner. A slice thickness of 2.0 mm was used for 126 patients, while 2.5 mm was used for the remaining 51 patients. ComBat harmonization was applied to reduce scanner-related variability, and all images were resampled to an isotropic voxel size of 1 mm^3^ to account for differences in slice thickness.

### 3.2. Classification Results

**Figure 2:**
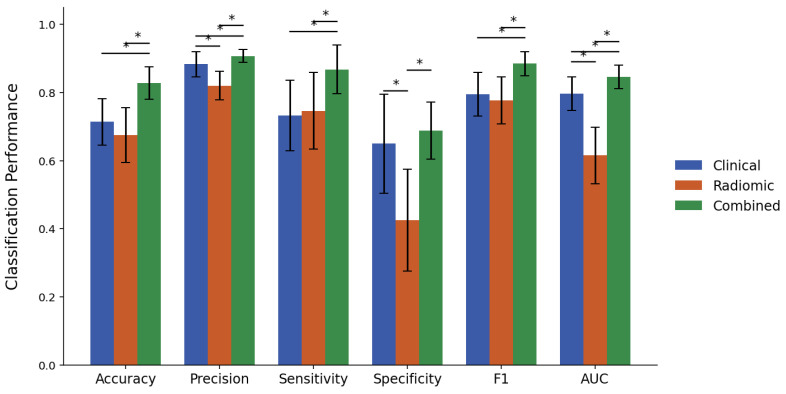
Performance metrics comparison of clinical, radiomic, and combined feature sets for predicting pCR vs. non-pCR (Criterion 1). Asterisks (*) denote statistically significant differences between feature sets (*p* < 0.05).

**Figure 3:**
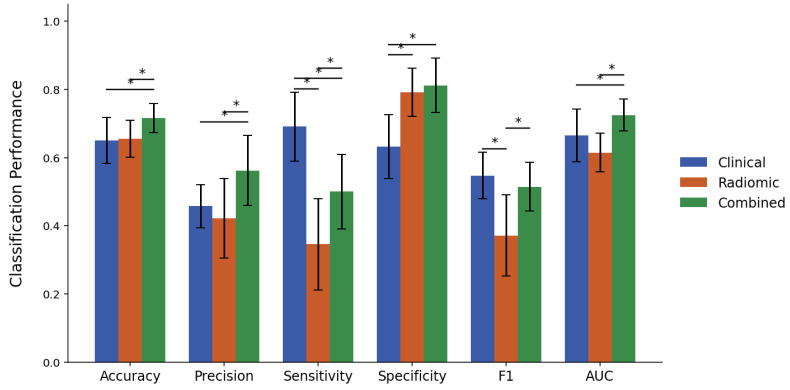
Performance metrics comparison of clinical, radiomic, and combined feature sets for predicting response vs. non-response (Criterion 2). Asterisks (*) denote statistically significant differences between feature sets (*p* < 0.05).

Figure 2 and Figure 3 present bar plots summarizing the classification performance of the XGBoost models developed using clinical, radiomic, and combined feature sets for response criteria 1 and 2, respectively, with the corresponding numerical values provided in Supplementary Tables S3 and S4. The corresponding p-values from paired two-tailed *t*-tests are reported in Supplementary Tables S5 and S6.

Under criterion 1, the clinical model achieved an accuracy of 71.4%, a precision of 88.3%, a sensitivity of 73.2%, a specificity of 65.0%, an F1-score of 0.795, and an AUC of 0.797. In contrast, the radiomic model exhibited statistically inferior performance with respect to precision (82.0%), specificity (42.5%), and AUC (0.615). The combined model yielded an accuracy of 82.8%, a precision of 90.8%, a sensitivity of 86.8%, a specificity of 68.8%, an F1-score of 0.885, and an AUC of 0.846. This combined model consistently outperformed the radiomic model on all measures and showed statistically significant improvement over the clinical model in every metric except specificity.

For criterion 2, the clinical model achieved an accuracy of 65.0%, a precision of 45.8%, a sensitivity of 69.1%, a specificity of 63.2%, an F1-score of 0.547, and an AUC of 0.666. The radiomic model exhibited greater specificity (79.2%) but underperformed in sensitivity (34.5%) and F1-score (0.371). The combined model again achieved superior results across most metrics, yielding an accuracy of 71.7%, a precision of 56.2%, a sensitivity of 50.0%, a specificity of 81.2%, an F1-score of 0.514, and an AUC of 0.725. Compared to the radiomic model, the combined model showed improvement across all metrics except specificity. Relative to the clinical model, the combined model demonstrated higher accuracy, specificity, and AUC, whereas its sensitivity was significantly lower, and the difference in F1-score was not statistically significant.

### 3.3. Features Selected

Based on the combined model, which demonstrated the best overall classification performance, the selection frequency of individual features across 10 data partitions was evaluated. Features selected at least five times are listed, along with their selection frequencies, in Table 3 and Table 4 for criteria 1 and 2, respectively.

As there were 177 patients in the cohort, 18 features were selected by mRMR, with subsequent RFE potentially further reducing the final number of selected features. Under criterion 1, an average of 12.6 features were selected per iteration, resulting in 35 distinct features identified across the 10 data partitions. Thirteen of these features appeared only once, while 22 were repeatedly selected across multiple iterations. Of these repeatedly selected features, 11 appeared in at least five iterations. This subset comprised five clinical factors and six radiomic features, which consisted of three intratumoral and three peritumoral features. Under criterion 2, the average number of selected features per iteration was 14.3, leading to a total of 31 unique features selected across all partitions. Seven features were selected in only a single iteration, while the remaining 24 were selected multiple times. Among these frequently selected features, 15 were chosen at least five times, consisting of seven clinical features and eight radiomic features. Within the radiomic frequently selected radiomic features, five were intratumoral and three were peritumoral features. Two features, (Intra) Shape_Sphericity and (Intra) GLCM_MaximalCorrelationCoefficient consistently selected across both criteria. Shape_Sphericity quantifies the roundness of the ROI relative to a perfect sphere, while GLCM_MaximalCorrelationCoefficient reflects the complexity of spatial relationships between voxel intensities within the ROI.

**Figure 4:**
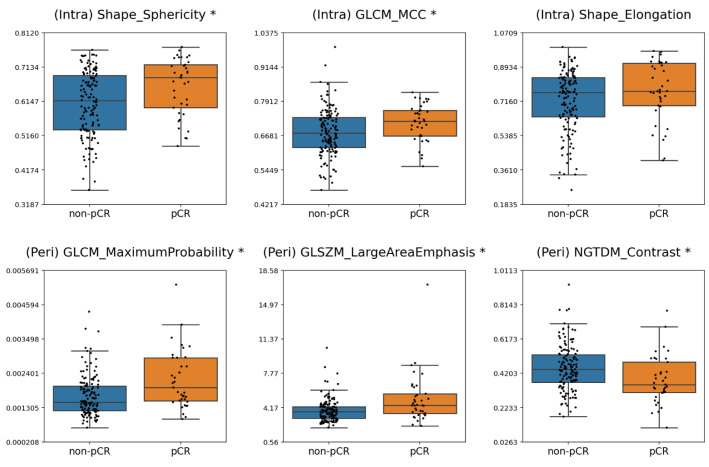
Box plots showing the frequently selected features under criterion 1. Significant differences in feature distributions between the pCR and non-pCR groups (*p* < 0.05) are indicated by an asterisk (*) beside the corresponding feature names. (MCC: Maximal Correlation Coefficient).

**Figure 5:**
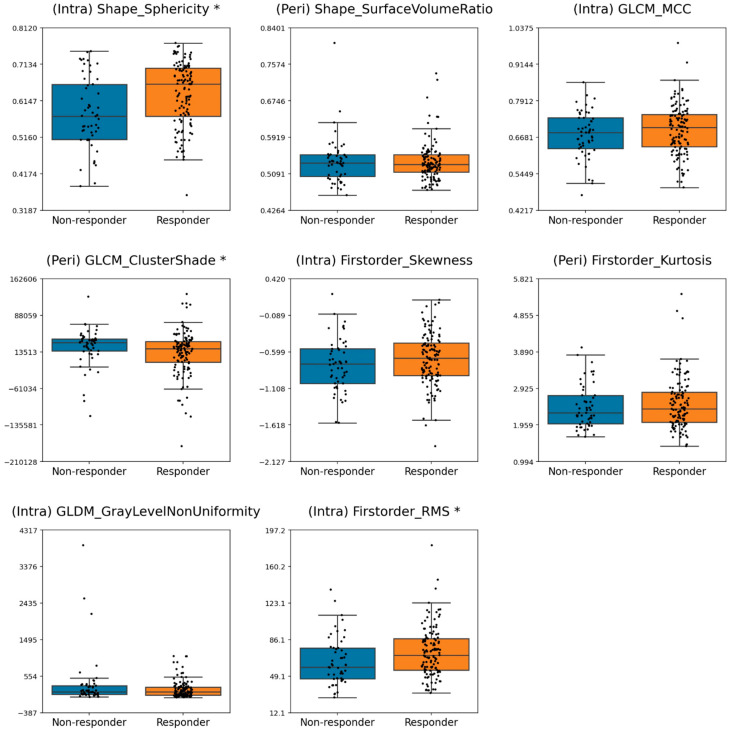
Box plots showing the frequently selected features under criterion 2. Significant differences in feature distributions between the response and non-response groups (*p* < 0.05) are indicated by an asterisk (*) beside the corresponding feature names. (MCC: Maximal Correlation Coefficient and RMS: Root Mean Square).

Figure 4 and Figure 5 present boxplots depicting the distribution of the frequently selected radiomic features for the responsive and non-responsive groups under criteria 1 and 2, respectively. The statistical significance of differences the feature distributions was also assessed. For criterion 1, significant differences between the pCR and non-pCR patients were identified for (Intra) Shape_Sphericity, (Intra) GLCM_MaximalCorrelationCoefficient, (Peri) GLCM_MaximumProbability, (Peri) GLSZM_LargeAreaEmphasis and (Peri) NGTDM_Contrast. For criterion 2, (Intra) Shape_Sphericity, (Peri) GLCM_ClusterShade, and (Intra) Firstorder_RootMeanSquare demonstrated statistically significant differences between responders and non-responders. Across both criteria, higher values of (Intra) Shape_Sphericity were significantly associated with response.

**Figure 6:**
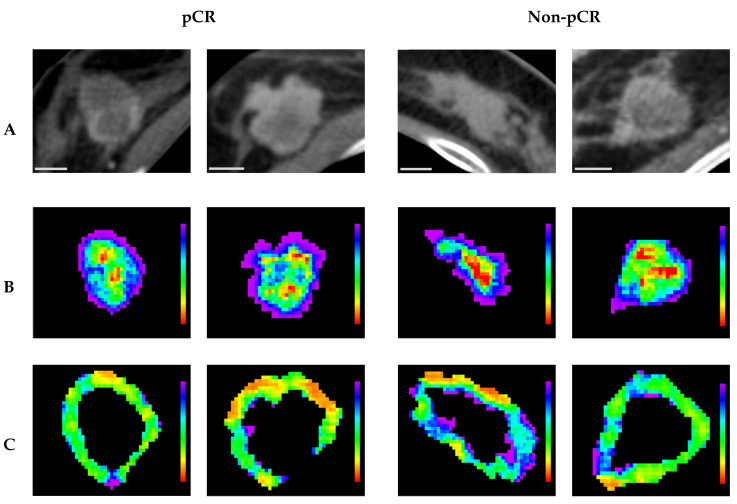
Representative pre-treatment CT images and parametric maps under criterion 1. Each column represents a different tumor associated with either pCR or non-pCR. (**A**) axial CT images; (**B**) parametric maps of (Intra) GLCM_MaximalCorrelationCoefficient; (**C**) parametric maps of (Peri) NGTDM_Contrast. A 10 mm scale bar is provided in the bottom-left corner of each CT image. Parametric maps are color-coded according to feature values, with (**B**) ranging from 0.7 (red) to 1 (purple) and (**C**) ranging from 0 (red) to 3 (purple).

**Figure 7:**
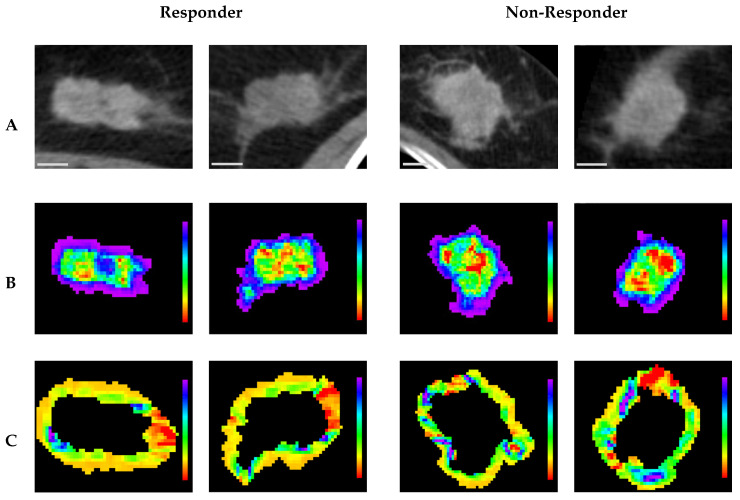
Representative pre-treatment CT images and parametric maps under criterion 2. Each column represents a different tumor associated with either a responder or non-responder. (**A**) axial CT images; (**B**) parametric maps of (Intra) GLCM_MaximalCorrelationCoefficient; (**C**) parametric maps of (Peri) GLCM_Clustershade. A 10 mm scale bar is provided in the bottom-left corner of each CT image. Parametric maps are color-coded according to feature values, with (**B**) ranging from 0.7 (red) to 1 (purple) and (**C**) ranging from −5000 (red) to 25,000 (purple).

Representative CT parametric maps for criteria 1 and 2 are presented in Figure 6 and Figure 7, respectively, illustrating spatial patterns of the selected features. Specifically, for (Intra) GLCM_MaximalCorrelationCoefficient, a feature consistently selected across both criteria, the non-responsive groups showed larger regions characterized by low feature intensity within the tumor core. This observation aligns with the box plots, which indicate lower GLCM_MaximalCorrelationCoefficient values in non-responsive groups. Under criterion 1, (Peri) NGTDM_Contrast maps in pCR cases revealed more homogeneous feature distributions characterized by lower overall values, whereas non-pCR cases displayed markedly greater heterogeneity, with generally higher feature intensities. Similarly, under criterion 2, (Peri) GLCM_ClusterShade maps for responders showed relatively homogeneous patterns with lower values, whereas non-responders exhibited greater spatial variability, highlighted by regions of higher intensity, predominantly green and blue. These spatial observations corresponded well with trends from the box plots, indicating lower feature values in responsive groups and higher values among non-responsive groups.

## 4. Discussion

This study demonstrates the feasibility of using radiomic features extracted from intratumoral and peritumoral segmentations of pre-treatment contrast-enhanced CT images, in combination with clinical variables, to predict the response to NAC in breast cancer patients. Recognizing the clinical significance of both complete and partial tumor responses, two separate response criteria were evaluated. The first criterion distinguished patients who achieved pCR from those who had residual tumors. The second criterion classified responses based on a reduction in tumor size greater than 30%, differentiating responders from non-responders. The latter group comprised patients whose disease remained stable or progressed despite NAC.

Analysis of clinical variables demonstrated that histologic grade and receptor status (ER, PR, and HER2) were significantly associated with treatment response under both criteria. Tumors with higher histologic grade, ER/PR negativity, and HER2 positivity demonstrated greater sensitivity to NAC. These variables are clinically well-established predictors of response, as anthracycline- and taxane-based regimens, which constitute the backbone of NAC for breast cancer, preferentially target aggressive tumors [1]. Histologic grade is a composite indicator of tumor aggressiveness, which evaluates mitotic count, nuclear pleomorphism, and tubule formation to reflect the proliferative and invasive potential of the cancer. Receptor status is an important biomarker that determines molecular subtypes and guides clinical decision-making. Tumors classified as Luminal A (ER/PR-positive, HER2-negative, low Ki-67) and Luminal B (ER/PR-positive, HER2-positive or negative, high Ki-67) are generally less responsive to NAC. In contrast, HER2-enriched (ER/PR-negative, HER2-positive) and triple-negative (ER/PR-negative, HER2-negative) subtypes are typically more chemosensitive. Furthermore, the availability of targeted therapy and immunotherapy enhances treatment efficacy in these aggressive subtypes. It should be noted that NAC response of HER2-enriched and triple negative subtypes is more strongly associated with improved survival outcome compared to the response of Luminal A and B subtypes [9]. For criterion 1, nodal status was significantly associated with pCR as well. Conversely, age and initial tumor size were not significantly associated with treatment response under either criterion. However, initial tumor size (*p* = 0.073) and age (*p* = 0.064) showed trends toward significance for criterion 1 and criterion 2, respectively.

To evaluate the additive predictive value of integrating CT-based radiomics with clinical information, a comparative analysis of clinical, radiomic, and combined feature model performance was conducted. Under Criterion 1, the clinical feature set resulted in an AUC of 0.797, which was significantly higher than the AUC of 0.615 obtained from the radiomic feature set. For criterion 2, the AUCs from the clinical and radiomic sets were 0.666 and 0.615, respectively, with no statistically significant difference. Integrating the radiomic features with the clinical features resulted in significant improvements in AUC, increasing to 0.846 for criterion 1 and 0.725 for criterion 2. The combined model also demonstrated complementary effects between clinical and radiomic features across sensitivity and specificity metrics. For criterion 1, while both clinical and radiomic models exhibited similar sensitivities of 73.2% and 74.6%, respectively, the clinical model exhibited notably higher specificity (65.0% versus 42.5%). Given that non-pCR was designated as the positive class in criterion 1, higher specificity suggests that pCR cases can be more effectively distinguished using clinical variables. For criterion 2, sensitivity was significantly higher for the clinical model (69.1%) than for the radiomic model (34.5%), while specificity was significantly higher for the radiomic model (79.2%) than for the clinical model (63.2%). Since non-response was designated as the positive class in criterion 2, the higher sensitivity of the clinical model indicates improved detection of non-responders from clinical variables, while the radiomic model aided in more accurate identification of responders. The combined feature set further enhanced overall classification performance, increasing sensitivity for criterion 1 to 86.8% and specificity for criterion 2 to 81.2%, both significantly higher than those achieved by the clinical model alone. For criterion 2, however, sensitivity decreased significantly from 69.1% in the clinical model to 50.0% in the combined model. This reduction was accompanied by an increase in precision from 45.8% to 56.2%, suggesting that although fewer non-responders were identified, those identified were classified with greater certainty. A similar decline in sensitivity for Criterion 2 using the combined feature set was observed in the previous work with MRI radiomics, although the difference in that case was not statistically significant [25]. Collectively, these findings suggest that although clinical features provide a strong predictive foundation, the addition of radiomic features can further refine model performance without compromising the baseline predictive value of clinical variables.

Model development and performance evaluation were conducted using ten independent splits of training and test sets. To avoid information leakage, feature selection was performed exclusively within the training set after partitioning, resulting in ten distinct feature sets and independent models. Across the ten iterations, variability in feature selection was observed, and the feature selection frequency was used as a surrogate for feature importance. For criteria 1 and 2, 11 and 15 features were selected in five or more iterations. Among the clinical features, the three receptor statuses were selected consistently. For criterion 1, PR and HER2 were selected in all ten partitions, while ER was selected in five. For criterion 2, HER2 and ER were each selected ten times, and PR was selected seven times. In addition, histologic grade was selected ten times under criterion 1 and eight times under criterion 2. Notably, discrepancies between statistical significance in univariate analysis and selection frequency were observed. In criterion 2, all seven clinical features were selected at least five times, although only the three receptor statuses and histologic grade exhibited statistical significance. Age, which only showed a trend toward significance (*p* = 0.064), was selected nine times, and nodal status was selected five times as well. While initial tumor size was chosen seven times under both criteria, it did not exhibit statistical significance in either. In machine learning, the predictive importance and statistical significance do not always correspond, as reflected in the present results [36].

For criterion 1 and criterion 2, seven and eight radiomic features, respectively, were selected in at least five of the ten data partitions. The frequently selected features for criterion 1 comprised three intratumoral features (Shape_Sphericity, Shape_Elongation, and GLCM_MaximalCorrelationCoefficient), and three peritumoral features (NGTDM_Contrast, GLCM_MaximumProbability, and GLSZM_LargeAreaEmphasis). For criterion 2, the frequently selected features included five intratumoral features (Shape_Sphericity, GLCM_MaximalCorrelationCoefficient, Firstorder_Skewness, GLDM_GrayLevelNonUniformity, and Firstorder_RootMeanSquared) and three peritumoral features (Shape_SurfaceVolumeRatio, GLCM_ClusterShade, and Firstorder_Kurtosis). Most notably, (Intra) Shape_Sphericity and (Intra) GLCM_MaximalCorrelationCoefficient were consistently associated with treatment response across both criteria. A more spherical tumor shape may indicate a less invasive morphology and reduced structural complexity, favoring homogeneous architecture and vascular perfusion, which can improve chemotherapy delivery and efficacy. Intratumoral radiomic features, such as GLCM_MaximalCorrelationCoefficient, capture spatial heterogeneity within the tumor, reflecting cell density, angiogenesis, and necrosis [37]. Highly vascularized tumors readily undergo vascular normalization, which can facilitate drug uptake and improve the likelihood of a chemotherapy response [38]. Peritumoral features are postulated to capture properties of the surrounding microenvironment. A previous study suggested that MRI-based peritumoral radiomics might reflect the presence of tumor-infiltrating lymphocytes [28], which are associated with better treatment outcomes [39]. However, the exact biological meaning of individual radiomic features is not yet fully understood, and future work integrating histopathological and molecular data with radiomic biomarkers will be essential for interpreting their significance.

Several prior studies have investigated CT-based radiomics for predicting NAC response in breast cancer. Moghadas-Dastjerdi et al. developed a model using intratumoral GLCM and second derivative texture features, achieving an AUC of 0.877 for distinguishing responders using leave-one-out cross-validation in a cohort of 72 patients [21]. Huang et al. proposed a radiomic nomogram incorporating clinical variables and both intratumoral and peritumoral features, which achieved an AUC of 0.818 for pCR prediction in a hold-out validation set of 77 patients [40]. Tsai et al. reported an AUC of 0.87 using a combination of clinical and CT-based radiomic features in a cohort of 329 patients, evaluated on an independent hold-out set [41]. Additionally, Moslemi et al. reported an accuracy of 75% for clinical-pathological response prediction using original texture features and wavelet features with leave-one-patient-out validation in 117 patients. While the predictive performance observed in this study is comparable to that of previous works, this study uniquely evaluates both pCR and clinical response and employs repeated evaluation across ten stratified partitions, providing a more robust assessment of generalizability. In addition to CT, other imaging modalities, such as ultrasound and MRI, have also been explored for NAC response prediction in breast cancer [19,25].

A key limitation of this study is that it relies on a single-institution dataset with a relatively small patient cohort, and no external validation has been performed. The use of ten independent test partitions, each serving as a distinct hold-out set, provides a rigorous and unbiased assessment of the model’s performance, partially mitigating overfitting. However, while this reduces the risk of accidental overfitting to one specific subset of the dataset, it does not eliminate the possibility of systematic biases unique to the data. Therefore, future studies should incorporate external validation using multi-center datasets with larger patient cohorts and variable image acquisition protocols. This will provide a more rigorous evaluation of the model’s robustness and applicability across diverse clinical settings. A larger dataset would also support multi-class classification, allowing differentiation between non-responders, partial responders, and complete responders, while maintaining sufficient sample sizes for each group. Additionally, increased cohort diversity would enable the development of subtype-specific models. Given that treatment response and prognosis vary across molecular subtypes, future work should consider stratified modeling approaches tailored to Luminal A, Luminal B, HER2-enriched, and triple-negative breast cancers. Moreover, future work should compare and combine radiomic features from CT with those derived from other imaging modalities available for breast cancer, such as MRI and QUS. This may help capture complementary biological information, provide a deeper understanding of imaging biomarkers, and lead to further improvements in classification performance. In addition, the absence of direct biological validation remains a major limitation in the interpretation of radiomic features. Although these features quantify heterogeneity associated with tumor biology, their direct biological relevance often remains uncertain. To address this limitation, future research should incorporate multimodal data, including histological analysis and molecular profiling, to identify meaningful biological correlates and improve the interpretability of radiomic signatures. Lastly, future studies should explore how predictive models based on radiomics can be integrated into clinical workflows to support treatment decision-making. This includes establishing thresholds for modifying treatment strategies and evaluating their clinical utility through prospective validation and cost–benefit analysis.

## 5. Conclusions

In conclusion, this study demonstrates the feasibility of predicting breast cancer response to NAC using machine learning models built from clinical information and pre-treatment CT imaging, both of which are routinely obtained as part of the standard diagnostic workup. The integration of intratumoral and peritumoral radiomic features with clinical variables improved model performance, leading to significantly better classification of pCR versus non-pCR and responders versus non-responders. These predictive models have the potential to support more personalized treatment strategies by enabling the early identification of patients who are less likely to benefit from standard NAC regimens, thereby guiding alternative therapeutic approaches before treatment initiation.

## Figures and Tables

**Table 1:** Comparison of clinical characteristics between pCR and non-pCR groups (Criterion 1).

Characteristics	pCR (N = 37)	Non-pCR (N = 140)	All (N = 177)	*p*-Value
Age (year)	50.2 ± 7.2	48.6 ± 10.8	48.9 ± 10.1	0.269
Initial Tumor Size (mm)	35.4 ± 16.1	42.0 ± 22.6	40.7 ± 21.6	0.073
Histologic Grade				0.003
I (%)	1 (2.7%)	9 (6.4%)	10 (5.6%)	
II (%)	8 (21.6%)	69 (49.3%)	77 (43.5%)	
III (%)	28 (75.7%)	62 (44.3%)	90 (50.8%)	
ER				<0.001
Negative (%)	24 (64.9%)	39 (27.9%)	63 (35.6%)	
Positive (%)	13 (35.1%)	101 (72.1%)	114 (64.4%)	
PR				<0.001
Negative (%)	30 (81.1%)	48 (34.3%)	78 (44.1%)	
Positive (%)	7 (18.9%)	92 (65.7%)	99 (55.9%)	
HER2				<0.001
Negative (%)	12 (32.4%)	103 (73.6%)	115 (65.0%)	
Positive (%)	25 (67.6%)	37 (26.4%)	62 (35.0%)	
Nodal Status				0.023
N0 (%)	11 (29.7%)	33 (23.6%)	44 (24.9%)	
N1 (%)	25 (67.6%)	79 (56.4%)	104 (58.8%)	
N2 (%)	0 (0%)	23 (16.4%)	23 (13.0%)	
N3 (%)	1 (2.7%)	5 (3.6%)	6 (3.4%)	

**Table 2:** Comparison of clinical characteristics between response and non-response groups (Criterion 2).

Characteristics	Response (N = 124)	Non-Response (N = 53)	All (N = 177)	*p*-Value
Age (year)	47.9 ± 9.2	51.3 ± 11.6	48.9 ± 10.1	0.064
Initial Tumor Size (mm)	41.5 ± 22.9	38.8 ± 18.0	40.7 ± 21.6	0.798
Histologic Grade				<0.001
I (%)	7 (5.6%)	3 (5.7%)	10 (5.6%)	
II (%)	43 (34.7%)	34 64.2%)	77 (43.5%)	
III (%)	74 (59.7%)	16 (30.2%)	90 (50.8%)	
ER				<0.001
Negative (%)	54 (43.5%)	9 (17.0%)	63 (35.6%)	
Positive (%)	70 (56.5%)	44 (83.0%)	114 (64.4%)	
PR				0.008
Negative (%)	63 (50.8%)	15 (28.3%)	78 (44.1%)	
Positive (%)	61 (49.2%)	38 (71.7%)	99 (55.9%)	
HER2				<0.001
Negative (%)	70 (56.5%)	45 (84.9%)	116 (65.2%)	
Positive (%)	54 (43.5%)	8 (15.1%)	62 (34.8%)	
Nodal Status				0.647
N0 (%)	33 (26.6%)	11 (20.8%)	44 (24.9%)	
N1 (%)	73 (58.9%)	31 (58.5%)	104 (58.8%)	
N2 (%)	14 (11.3%)	9 (17.0%)	23 (13.0%)	
N3 (%)	4 (3.2%)	2 (3.8%)	6 (3.4%)	

**Table 3:** Summary of frequently selected features for criterion 1. Features are categorized into clinical (white), intratumoral radiomic (blue), and peritumoral radiomic (yellow) groups.

Features	#
PR	10
(Intra) Shape_Sphericity	10
HER2	10
Histologic Grade	10
(Intra) GLCM_MaximalCorrelationCoefficient	9
(Intra) Shape_Elongation	8
Initial Tumor Size (mm)	7
(Peri) GLCM_MaximumProbability	5
(Peri) GLSZM_LargeAreaEmphasis	5
ER	5
(Peri) NGTDM_Contrast	5

**Table 4:** Summary of frequently selected features for criterion 2. Features are categorized into clinical (white), intratumoral radiomic (blue), and peritumoral radiomic (yellow) groups.

Features	#
HER2	10
(Intra) Shape_Sphericity	10
ER	10
(Peri) Shape_SurfaceVolumeRatio	9
Age	9
Histologic Grade	8
(Intra) GLCM_MaximalCorrelationCoefficient	8
(Peri) GLCM_ClusterShade	8
PR	7
Initial Tumor Size (mm)	7
(Intra) Firstorder_Skewness	6
(Peri) Firstorder_Kurtosis	6
Nodal Status	5
(Intra) GLDM_GrayLevelNonUniformity	5
(Intra) Firstorder_RootMeanSquared	5

## Data Availability

Data are available upon request (contact the Czarnota Lab at Sunnybrook Health Sciences Centre).

## Supplementary Materials

The following supporting information can be downloaded at: https://www.mdpi.com/article/10.3390/cancers17162706/s1. Table S1. List of radiomic features extracted using PyRadiomics Table S2. Hyperparameter tuning settings for XGBoost machine learning. Table S3. Performance metrics of clinical, radiomic, and combined feature sets for predicting pCR vs. non-pCR (Criterion 1). Table S4. Performance metrics of clinical, radiomic, and combined feature sets for predicting response vs. non-response (Criterion 2). Table S5. *p*-values of two-tailed *t*-test comparing classification performance across the three models for criterion 1. Statistical significance with *p* < 0.05 is marked with *, and *p* < 0.001 is marked with **. Table S6. *p*-values of two-tailed *t*-test comparing classification performance across the three model sets for criterion 2. Statistical significance with *p* < 0.05 is marked with *, and *p* < 0.001 is marked with **.
