# Supplementary material for: A Priori Prediction of Breast Cancer Response to Neoadjuvant Chemotherapy Using CT Radiomics
Source: Cancers (Basel). 2025 Aug 20;17(16):2706. doi: 10.3390/cancers17162706 (PMC12384108; doi:10.3390/cancers17162706)
Supplement: Supplementary file 1 [file cancers-17-02706-s001.zip › cancers-3751975-supplementary.pdf]

Table S1. List of radiomic features extracted using PyRadiomics

Feature Class	Feature Number	Feature Name
**3D Shape**	14	Elongation
		Flatness
		Least Axis Length
		Major Axis Length
		Maximum 2D Diameter Column
		Maximum 2D Diameter Row
		Maximum 2D Diameter Slice
		Maximum 3D Diameter
		Mesh Volume
		Minor Axis Length
		Sphericity
		Surface Area
		Surface Volume Ratio
		Voxel Volume
**First Order**	18	10 Percentile
		90 Percentile
		Energy
		Entropy
		Interquartile Range
		Kurtosis
		Maximum
		Mean Absolute Deviation
		Mean
		Median
		Minimum
		Range
		Robust Mean Absolute Deviation
		Root Mean Squared
		Skewness
		Total Energy
		Uniformity
		Variance
**Gray Level Co-occurrence Matrix (GLCM)**	24	Autocorrelation
		Joint Average
		Cluster Prominence
		Cluster Shade
		Cluster Tendency
		Contrast
		Correlation

		Difference Average<br>Difference Entropy<br>Difference Variance<br>Joint Energy<br>Joint Entropy<br>Informational Measure of Correlation (IMC) 1<br>Informational Measure of Correlation (IMC) 2<br>Inverse Difference Moment (IDM)<br>Inverse Difference Moment Normalized (IDMN)<br>Inverse Difference (ID)<br>Inverse Difference Normalized (IDN)<br>Maximal Correlation Coefficient (MCC)<br>Inverse Variance<br>Maximum Probability<br>Sum Average<br>Sum Entropy<br>Sum Squares
**Gray Level Run Length Matrix (GLRLM)**	16	Gray Level Non Uniformity<br>Gray Level Non Uniformity Normalized<br>Gray Level Variance<br>High Gray Level Run Emphasis<br>Long Run Emphasis<br>Long Run High Gray Level Emphasis<br>Long Run Low Gray Level Emphasis<br>Low Gray Level Run Emphasis<br>Run Entropy<br>Run Length Non Uniformity<br>Run Length Non Uniformity Normalized<br>Run Percentage<br>Run Variance<br>Short Run Emphasis<br>Short Run High Gray Level Emphasis<br>Short Run Low Gray Level Emphasis
**Gray Level Size Zone Matrix (GLSZM)**	16	Gray Level Non Uniformity<br>Gray Level Non Uniformity Normalized<br>Gray Level Variance<br>High Gray Level Zone Emphasis<br>Large Area Emphasis<br>Large Area High Gray Level Emphasis<br>Large Area Low Gray Level Emphasis<br>Low Gray Level Zone Emphasis<br>Size Zone Non Uniformity<br>Size Zone Non Uniformity Normalized

		Small Area Emphasis<br>Small Area High Gray Level Emphasis<br>Small Area Low Gray Level Emphasis<br>Zone Entropy<br>Zone Percentage<br>Zone Variance
**Gray Level Dependence Matrix (GLDM)**	14	Dependence Entropy<br>Dependence Non Uniformity<br>Dependence Non Uniformity Normalized<br>Dependence Variance<br>Gray Level Non Uniformity<br>Gray Level Variance<br>High Gray Level Emphasis<br>Large Dependence Emphasis<br>Large Dependence High Gray Level Emphasis<br>Large Dependence Low Gray Level Emphasis<br>Low Gray Level Emphasis<br>Small Dependence Emphasis<br>Small Dependence High Gray Level Emphasis<br>Small Dependence Low Gray Level Emphasis
**Neighbouring Gray Tone Difference Matrix (NGTDM)**	5	Busyness<br>Coarseness<br>Complexity<br>Contrast<br>Strength

Table S2. Hyperparameter tuning settings for XGBoost machine learning

Hyperparameter	Value
learning_rate	0.01, 0.05, and 0.1
eval_metric	auc
max_depth	3, 5, and 7
min_child_weight	1, 3, 5, and 7
n_estimators	100, 250, and 500

Table S3. Performance metrics of clinical, radiomic, and combined feature sets for predicting pCR vs. non-pCR (Criterion 1).

Feature Set	Accuracy (%) ± SD	Precision (%) ± SD	Sensitivity (%) ± SD	Specificity (%) ± SD	F1 ± SD	AUC ± SD
Clinical	71.4 ± 6.8	88.3 ± 3.7	73.2 ± 10.4	65.0 ± 14.6	0.795 ± 0.065	0.797 ± 0.049
Radiomic	67.5 ± 8.1	82.0 ± 4.2	74.6 ± 11.2	42.5 ± 15.0	0.777 ± 0.068	0.615 ± 0.083
Combined	82.8 ± 4.8	90.8 ± 1.9	86.8 ± 7.2	68.8 ± 8.4	0.885 ± 0.035	0.846 ± 0.034

Table S4. Performance metrics of clinical, radiomic, and combined feature sets for predicting response vs. non-response (Criterion 2).

Feature Set	Accuracy (%) ± SD	Precision (%) ± SD	Sensitivity (%) ± SD	Specificity (%) ± SD	F1 ± SD	AUC ± SD
Clinical	65.0 ± 6.7	45.8 ± 6.3	69.1 ± 10.1	63.2 ± 9.4	0.547 ± 0.069	0.666 ± 0.077
Radiomic	65.6 ± 5.4	42.1 ± 11.7	34.5 ± 13.4	79.2 ± 7.1	0.371 ±0.119	0.615 ±0.057
Combined	71.7 ± 4.3	56.2 ± 10.3	50.0 ± 10.9	81.2 ± 8.0	0.514 ± 0.071	0.725 ± 0.047

Table S5. p-values of two-tailed t-test comparing classification performance across the three models for criterion 1. Statistical significance with $p < 0.05$ is marked with * and $p < 0.001$ is marked with **

Comparison	Accuracy	Precision	Sensitivity	Specificity	F1	AUC

Clinical / Radiomic	0.356	*	0.798	*	0.617	**
Clinical / Combined	*	*	*	0.343	*	*
Radiomic / Combined	*	**	*	**	*	**

Table S6. *p*-values of two-tailed t-test comparing classification performance across the three models sets for criterion 2. Statistical significance with $p < 0.05$ is marked with * and $p < 0.001$ is marked with **

Comparison	Accuracy	Precision	Sensitivity	Specificity	F1	AUC
Clinical / Radiomic	0.804	0.350	**	**	*	0.126
Clinical / Combined	*	*	*	*	0.329	*
Radiomic / Combined	*	*	*	0.504	**	**
